# Anomalous Origin of the Left Main Coronary Artery From the Right Sinus of Valsalva With an Intramural Course: A Rare Cause of Cardiac Arrest During Routine Patent Ductus Arteriosus Device Closure

**DOI:** 10.7759/cureus.93897

**Published:** 2025-10-05

**Authors:** Mary Teddy Akech, Twalib Aliku, Bernard Obongonyinge, Nestor Mbabazi, Sulaiman Lubega

**Affiliations:** 1 Paediatric Cardiology, Uganda Heart Institute, Kampala, UGA; 2 Paediatric Cardiology, Uganda Christian University School of Medicine, Mukono, UGA; 3 Paediatric Cardiology, Mulago National Referral Hospital, Kampala, UGA

**Keywords:** anomalous coronary artery, cardiac arrest, left main coronary artery, patent ductus arteriosus, pda device closure, pediatric cardiac catheterization

## Abstract

Congenital coronary artery anomalies (CCAAs) are rare. Usually, the myocardium derives its blood supply from the left and right main coronary arteries that arise from the left and right aortic sinuses of Valsalva respectively. However, in rare circumstances the left main coronary artery (LMCA) may have an anomalous origin from the right aortic sinus of Valsalva (RASV). The LMCA may take any of the following anomalous pathways: posterior to the aorta, anterior to the right ventricular outflow tract and between the aorta and the right ventricular outflow tract (RVOT).

The anomalous course of the LMCA between the aorta and RVOT is sometimes called a "suicide coronary artery". It has been associated with sudden cardiac death during or just after vigorous exercise due to myocardial ischemia and development of ventricular arrhythmias. Although the first presentation of the anomaly is sometimes sudden cardiac death, in many of these patients there may be a history of syncope or prolonged chest pain before the fatal event. However, some patients are asymptomatic until the fatal event.

This case highlights a rare occurrence of cardiac arrest in a six-year-old female undergoing routine patent ductus arteriosus (PDA) device closure. She was later diagnosed with a "suicide coronary" (anomalous left main coronary artery origin from the right aortic sinus of Valsalva).

## Introduction

The prevalence of congenital coronary artery anomalies (CCAAs) is about 1-2% among patients undergoing cardiac catheterization [1,2]. Anomalous origin of coronary artery (AOCA) from a contralateral aortic sinus are more prevalent (about 1%) than other coronary anomalies [1]. The anomalies of right coronary artery (RCA) origin from the contralateral aortic sinus are more often seen than that of the left coronary artery (LCA) [1,2].

Anomalous left main coronary artery (LMCA) from the right aortic sinus of Valsalva (RASV) may take four main pathways to reach its designated territory of supply [3]. These include: posterior to the aorta, anterior to the right ventricular outflow tract, within the ventricular septum beneath the right ventricular infundibulum or between the aorta and the right ventricular outflow tract (RVOT) [3]. The anomalous course of the LMCA between the aorta and RVOT is sometimes called a "suicide coronary artery". It has been associated with sudden cardiac death during or just after vigorous exercise [3,4]. 

Sudden cardiac death is a result of myocardial ischemia and development of lethal ventricular arrhythmias. The pathophysiological mechanisms of sudden cardiac death may include coronary ostial stenosis, acute angle of take-off, compression of the inter-arterial segment between the aorta and pulmonary artery, compression of an intramural segment during exercise, and obstruction by a flap-like ridge related to an acutely angulated coronary artery [3,5]. Furthermore, in response to increased myocardial oxygen demand during exercise, the aortic root becomes distended during cardiac systole, resulting in compression of the anomalous LMCA artery [5]. Manipulations in the RVOT during right heart catheterization procedures may also lead to compression of the anomalous LMCA from the right aortic sinus of Valsalva.

Clinical presentation is common among adolescents, young adults or athletes during exercise. The first sign of the anomaly is sometimes sudden death or a fatal myocardial infarction. In many of these patients, there may be a history of syncope or prolonged chest pain before the fatal event. However, some patients are asymptomatic until the fatal event [5].

Given that the first presentation may be sudden cardiac death in most patients with AOCA, clinical evaluation is often challenging. Symptoms of chest pain, shortness of breath and syncope during exertion should prompt a thorough evaluation [6]. In those who have had myocardial infarction, there may be symptoms of heart failure. Physical examination is generally normal in asymptomatic patients. The electrocardiograph (ECG) is usually normal in the absence of ischemia. Echocardiography can demonstrate an anomalous origin of a coronary artery. It requires a high index of suspicion. However, it has limitations in older children because of poor acoustic window. Computed tomography (CT) and magnetic resonance (MR) coronary angiography are the standard tests for evaluation of coronary artery anomalies [6]. Functional studies such as exercise tolerance test can be performed to assess myocardial perfusion at rest and during stress [6]. 

In this report we describe a case of a six-year-old female who was diagnosed with an anomalous left main coronary artery origin from the right aortic sinus of Valsalva with an intra-mural course (suicide coronary artery) following cardiac arrest during a routine patent ductus arteriosus (PDA) device closure.

PDA is the second most common congenital heart defect (CHD) among infants and children. PDAs can be classified by size into very small (<1.5 mm), small (1.5-3.0 mm), moderate (3-5 mm), and large (>5 mm). Moderate and large PDAs and those with significant systemic to pulmonary shunting pose a greater risk for complications like heart failure and pulmonary hypertension. Most often, PDAs are amenable to transcatheter device closure, an effective and less invasive method of treatment [7].

PDA can be associated with, or a component of many other CHDs but rarely with CCAAs [8]. Although CCAAs are known to be associated with some CHDs such as tetralogy of Fallot and truncus arteriosus, it is thought to be rare with isolated PDA [8].

Adverse events including cardiac arrest are rare during pediatric cardiac catheterization. Particularly for PDA device closure, the rates are significantly lower compared to other interventional cases [9]. Additionally, while congenital coronary anomalies are a common cause of cardiac arrest in adolescents and young adults, it is less frequent in children undergoing PDA closure [9].

## Case presentation

A six-year-old female was referred to the Uganda Heart Institute (UHI) for PDA device closure. Uganda Heart Institute is the only National Referral center for pediatric cardiac catheterization. She had been diagnosed at the age of two years with symptoms of poor weight gain, exertional dyspnea and recurrent respiratory illnesses but was subsequently lost to follow-up. She had had four admissions for pneumonia in the previous five years. The general exam was unremarkable. Her weight was 21.7kg and height was 122cm (weight for height z-score is >-1SD); this falls in the 10th to 25th centile for weight-for-height, which is normal for her age.

The vital signs were all normal, with heart rate = 89 beats per minute, blood pressure (BP) = 99/56mmHg, peripheral capillary oxygen saturation (SPO2) = 98%, and respiratory rate 29 breaths per minute. Cardiac exam was significant for left precordial bulge, apical heave and a continuous murmur in the left upper sternal border. The complete blood count, renal function and electrolytes were normal (Table 1). The liver function tests were unremarkable. Serologies for HIV, hepatitis B and hepatitis C were all negative. The blood group was A rhesus D positive. Cardiac markers creatinine kinase (CK)-MB and troponin I done after the cardiac arrest were unremarkable.

**Table 1:** Results of serial blood tests performed on the patient before and after the procedures. WBC- white blood cells; NEUT- neutrophils, Lym- lymphocytes; Hb- Hemoglobin; PLT- platelets; CRP- C-reactive protein; Cr- creatinine; Ur- urea; K- potassium, Na- sodium; Cl- chloride. Other tests were normal hence not included in the table.

Parameter	Reference range	Unit	Pre-procedure	Post-procedure (ICU)	Post-procedure (at discharge)
WBC	4.00 - 12.00	10^9/L	6.48	7.78	4.84
NEUT	2.00 - 8.00	10^9/L	3.88	5.16	1.24
LYM	0,80 - 7.00	10^9/L	2.26	2.02	2.97
Hb	12.0 - 16.0	g/dl	13.1	13.6	13.4
PLT	100 - 300	10^9/L	206	235	240
CRP	0.01 - 0.28	mg/dl	0.46	2.59	0.1
Cr	0.20 - 0.80	mg/dl	0.51	0.44	0.41
Ur	0.0 - 50.0	mg/dl	13.5	18.4	16.4
K	3.50 - 5.50	mmol/l	3.57	3.88	3.52
Na	132.0- 153.0	mmol/l	136.9	138.5	138.3
Cl	98.0 - 114.0	mmol/l	105.7	102.8	103.4

**Figure 1:**
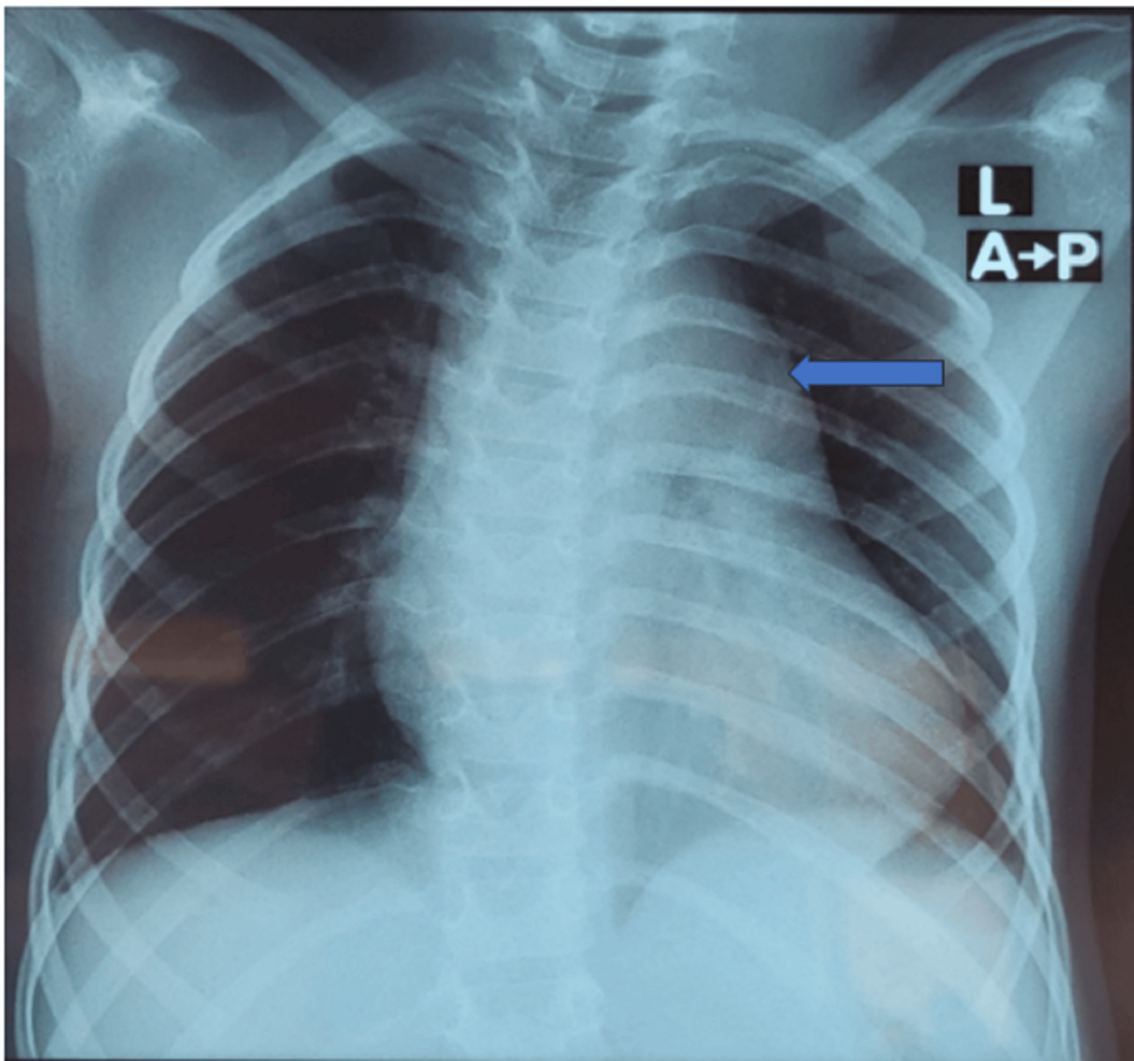
Chest radiograph showing cardiomegaly, dilated main pulmonary artery (blue arrow)

**Figure 2:**
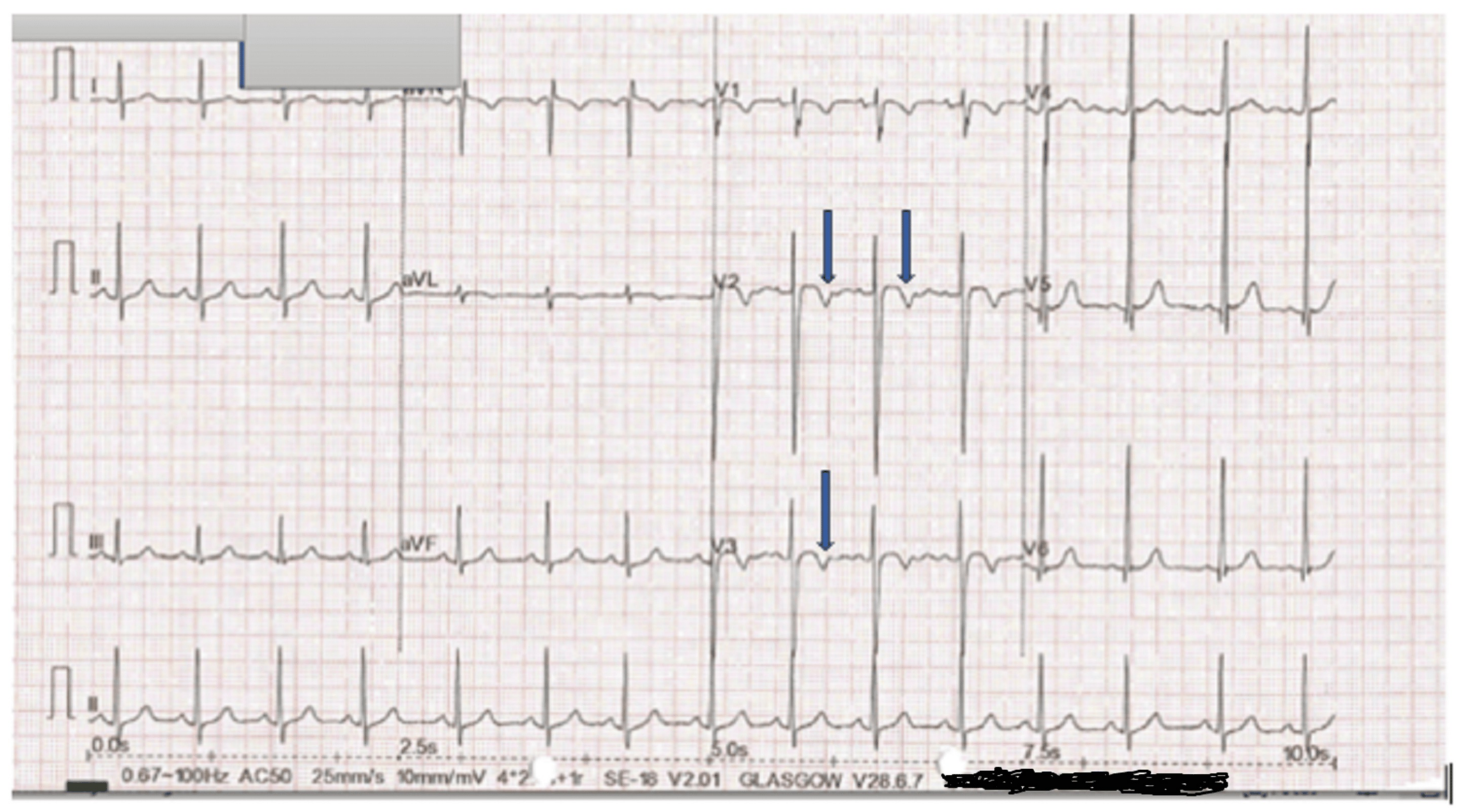
Pre-operative electrocardiograph (ECG) showing sinus rhythm with juvenile T-wave pattern (arrows)

**Figure 3:**
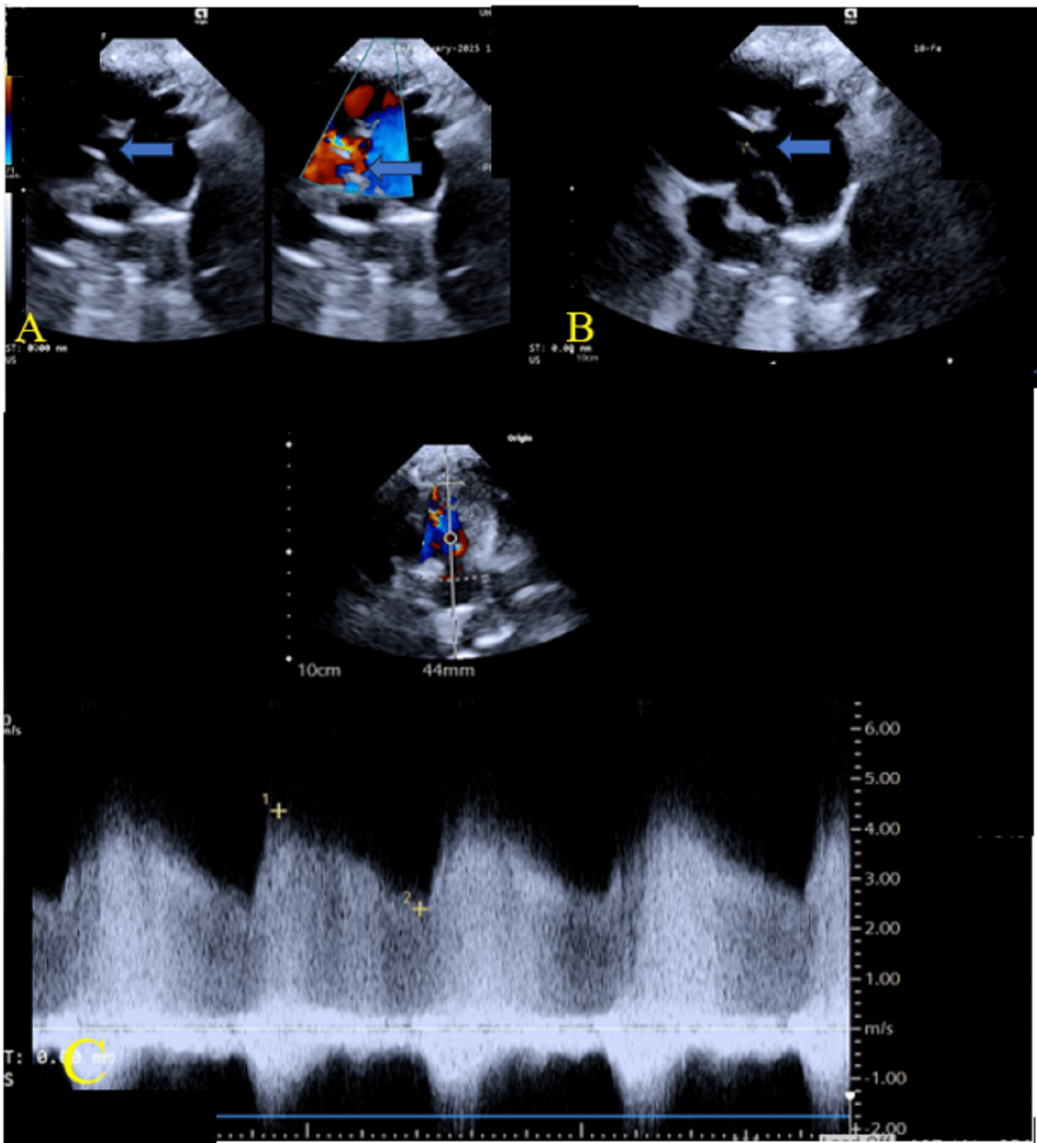
Transthoracic echocardiogram demonstrating a moderately sized short conical 5mm patent ductus arteriosus (PDA) with a left-to-right shunt. A: 2D and color Doppler (arrow). B: PDA measuring 5mm (arrow). C: Continuous-wave (CW) Doppler showing continuous flow.

The chest radiograph (Figure 1) showed a dilated main pulmonary artery (MPA). This is consistent with diagnosis of a moderately sized PDA. The electrocardiograph (ECG) (Figure 2) done prior to the procedure showed sinus rhythm with juvenile T-wave pattern, which is normal for her age and race. There were no features of myocardial ischemia on the ECG. The transthoracic echocardiogram (TTE) demonstrated a moderately sized short conical 5mm PDA with a left-to-right shunt (Figure 3). She was scheduled for an elective PDA device closure. Note that although assessing the coronary anatomy is part of our routine first TTE protocol, the coronary anomaly was missed in the initial evaluation of this child.

Procedure

**Figure 4:**
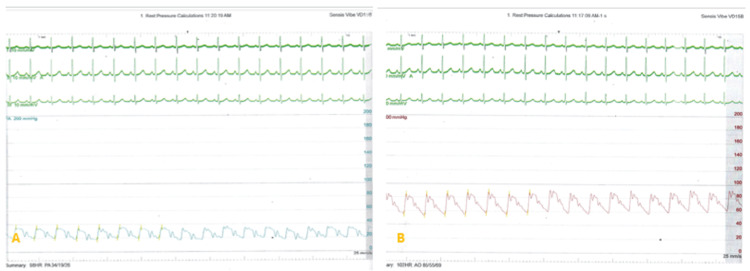
Catheterization tracings. A: Baseline pulmonary artery pressure of 34/19 (mean 26) mmHg. Notice ECG showing sinus rhythm; B: Baseline aortic pressure of 85/55 (mean 69) mmHg.

**Figure 5:**
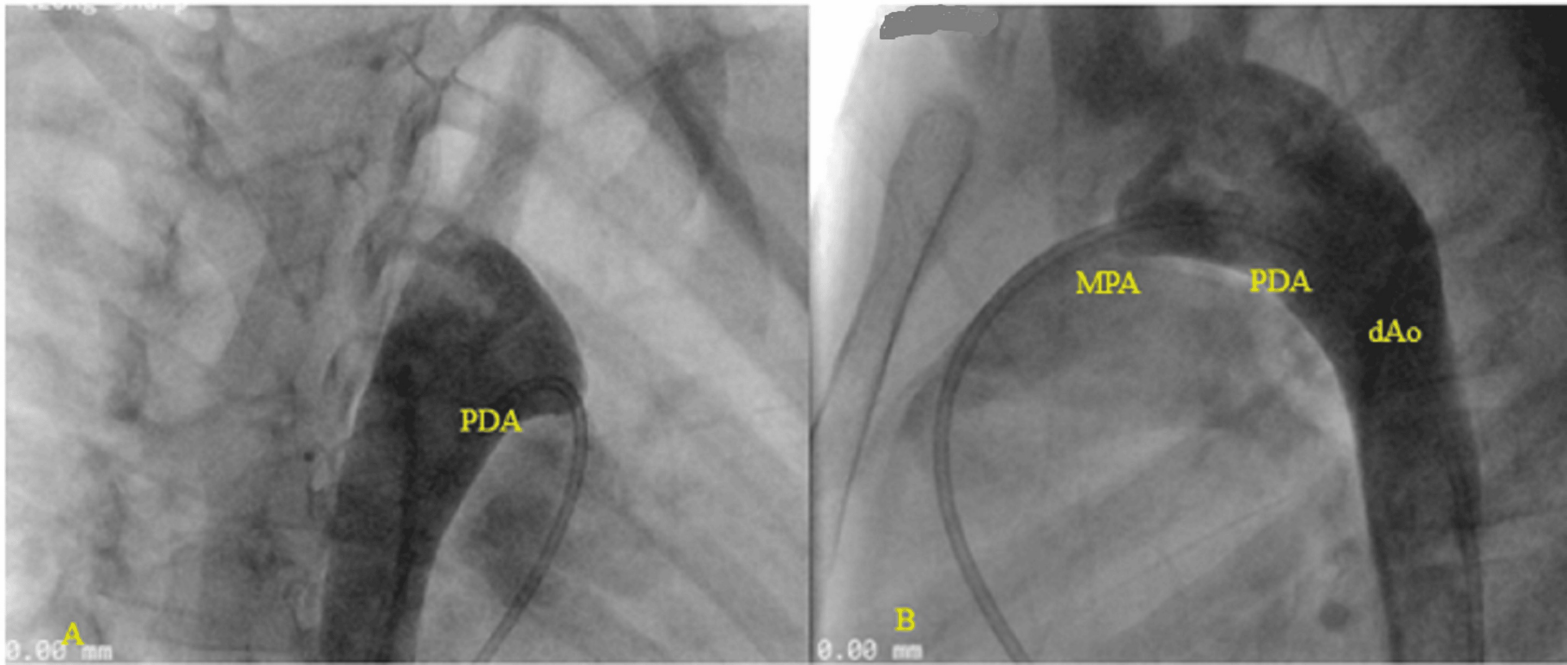
Cardiac catheterization; descending aorta angiogram demonstrating a short conical 5mm with good ampulla. A: antero-posterior view, B: lateral view PDA: patent ductus arteriosus, MPA: main pulmonary artery, dAo: descending aorta

**Figure 6:**
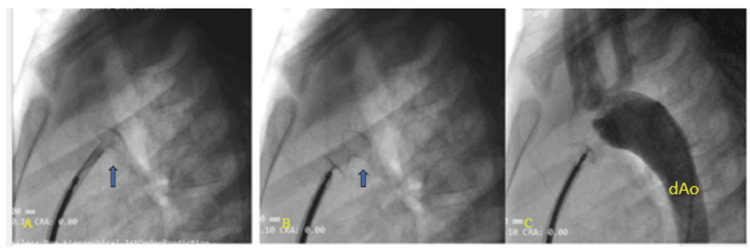
Cardiac catheterization during patent ductus arteriosus (PDA) device closure. A: the aortic disc deployed, B: pulmonary disc deployed, C: check angiogram revealing good device placement with no residual shunt dAo: descending aorta

**Figure 7:**
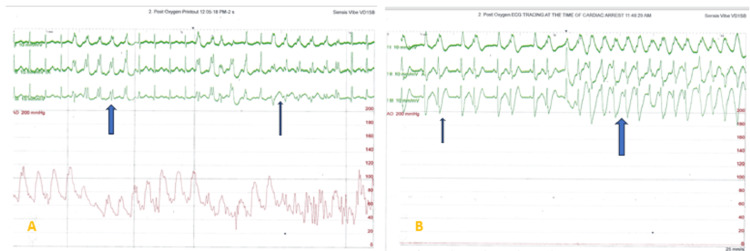
A: Notice the episodes of VT (thick arrows) with ST elevation (thin arrow) on the ECG tracing; B: ECG tracing showing runs of ventricular bigeminy (thin arrow) before VT

**Figure 8:**
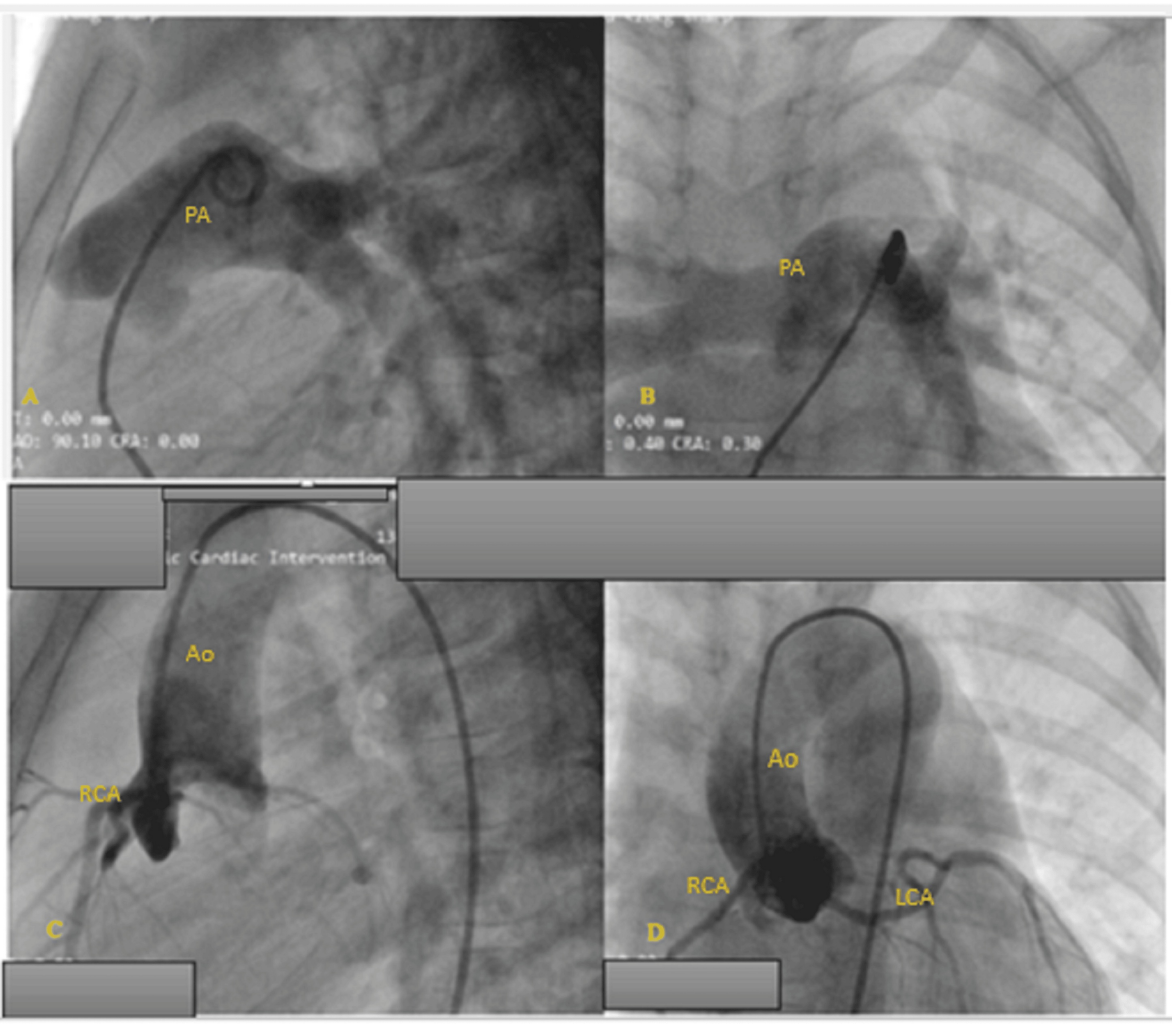
PA and aortic root angiogram. A and B: normal pulmonary angiogram. C and D: ascending aorta angiogram - both coronary arteries arise from the right aortic sinus of Valsalva, the LMCA coursing between the PA and the aorta. Ao: aorta, PA: pulmonary artery, LCA: left coronary artery, RCA: right coronary artery, LMCA: left main coronary artery

The catheterization procedure was performed under conscious sedation with double right femoral access. The pre-procedure pressure measurements were as follows: pulmonary artery pressure: 34/19 (mean 26) mmHg; descending aorta pressure: 85/55(mean 69) mmHg (Figure 4). A descending aortogram performed using a 5F NIH catheter demonstrated a short conical PDA measuring 5mm with a good ampulla (Figure 5). A decision was made to close the duct using an 8/6 Occlutech duct occluder device. The PDA was crossed antegrade using a combination of multipurpose catheter (MPA-2) and a glide wire. This was then exchanged for a Teflon wire through which a 7F Mullins sheath was advanced through the wire. We proceeded to deliver the device as per standard practice and deployed the aortic disc (Figure 6A). A check angiogram revealed good device placement with no residual shunt. The patient remained stable throughout this period. When the pulmonary disc was deployed (Figure 6B, 6C), we noted a paradoxical drop in the descending aorta pressure (systolic BP 60mmHg), followed by a continued drop in the heart rate (to 50 bpm), ST segment elevation and then a pulseless ventricular tachycardia (cardiac arrest) (see tracings in Figure 7). At this point we started chest compressions and gave adrenaline. Simultaneously, the device was retrieved into the delivery sheath and the whole system pulled into the inferior vena cava (IVC). Immediately, we noted a return to sinus rhythm and an improvement in the descending aorta pressure. We suspected a coronary artery anomaly and proceeded to do pulmonary artery (Figure 8A, 8B), and aortic root angiograms. The main pulmonary angiogram did not show any anomalous coronary from the coronary artery (ALCAPA). The ascending aorta angiogram revealed both coronary arteries arising from the right aortic sinus of Valsalva through separate ostia, with the LMCA coursing between the pulmonary artery and the aorta (Figure 8C, 8D).

**Figure 9:**
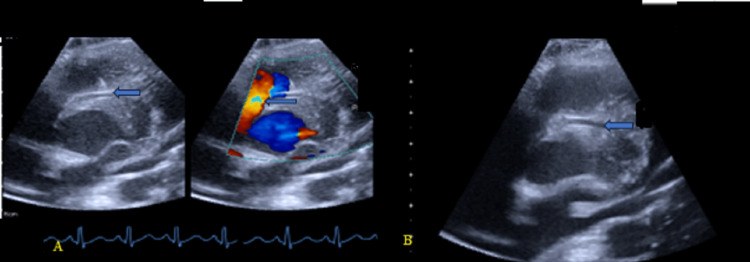
Repeat transthoracic echocardiography demonstrating the anomalous origin of the LMCA from the right aortic sinus. A: 2D and color, B: 2D (blue arrow) LMCA: left main coronary artery

The catheterization procedure was aborted. We presumed that the device and its delivery system were compressing the anomalous left main coronary artery coursing between the pulmonary artery and the aorta thus compromising myocardial perfusion. The patient was taken to the operating theatre on the same day for PDA surgical ligation. The operating theatre is just adjacent to the catheterization laboratory, which eased patient transfer. Surgery was done successfully. During a repeat TTE (Figure 9) done after the PDA ligation, the anomalous origin of the LMCA from the right aortic sinus could be demonstrated by slightly rotating the transducer clockwise and to the left of the patient from the standard parasternal short-axis view. CT coronary angiography was requested for but the family was unable to do it due to costs. Our hospital does not have in-house CT or MR imaging services; it is outsourced and usually very costly. 

She spent a total of six days in hospital, two of which were in ICU. She improved and was discharged home in good general condition. An exercise tolerance test was not done on this admission. The child and the family were counselled about restriction of vigorous and high-intensity activities since she is at high risk of myocardial ischemia and sudden cardiac death. The family was also counselled about the need for coronary artery translocation surgery, which is not currently possible in our country. She has had three follow-up visits since discharge and remains in good general condition, with no symptoms pointing to myocardial infarction or ischemia.

## Discussion

Children with AOCA from the contralateral aortic sinus do not always present with features of myocardial ischemia. This may be partly due to their levels of physical activity, presence of collateral supply or the specific anatomical course of the anomalous artery, particularly if the anomalous branch does not course between the aorta and the RVOT [4,6,10,11]. Although the first sign of the anomaly is sometimes sudden death or a fatal myocardial infarction, in many of these patients there may be a history of syncope or prolonged chest pain before the fatal event [6,11]. The case in discussion, however, didn’t have prior history of syncope or chest pain. She didn’t have symptoms pointing to her coronary abnormality but more to heart failure due to the PDA. 

Given that the first presentation may be sudden cardiac arrest in most patients with AOCA, clinical evaluation is often challenging. Symptoms of chest pain, shortness of breath and syncope during exertion should prompt a thorough evaluation [6, 10]. Physical exam and ECG are usually normal in most patients. As seen in our case, the initial symptoms and signs of the child were attributed to heart failure due to the presence of a moderately sized PDA. Her ECG did not show features of ST-segment depression in leads V5, V6, I and AVL.

Echocardiography can demonstrate an anomalous origin of a coronary artery, but requires a high index of suspicion, and has limitations in older children due to poor acoustic windows [6,12]. The echo findings in this child showed a PDA, with no features of ischemia such as regional wall motion abnormalities, left ventricular dysfunction, subendocardial fibrosis or mitral valve regurgitation. The AOCA diagnosis was missed in the initial echocardiography evaluation. The presence of a major cardiac anomaly, a PDA in this case, probably shifted the focus away from completing a comprehensive first echocardiography study in this patient. A CT or MR coronary angiography is the standard test for evaluation of coronary artery anomalies [6,10]; however, our hospital doesn't have these services in-house, and outsourcing is very costly. Echocardiography is more readily accessible. Functional studies such as exercise tolerance test can be performed to assess myocardial perfusion at rest and during stress [6]. In this case, functional studies were not done because the child had experienced a major cardiac event during the catheterization procedure.

**Figure 10:**
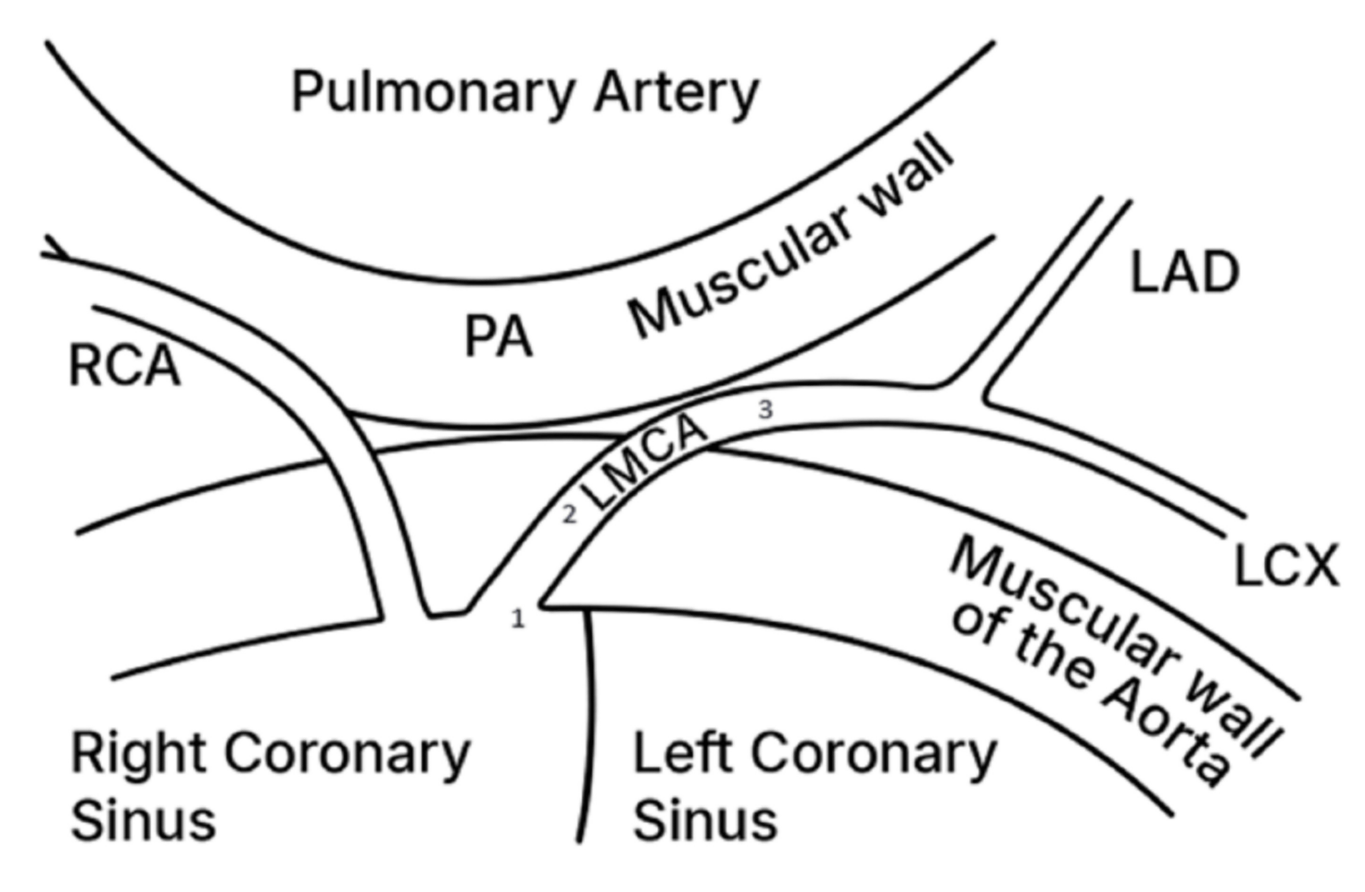
Anomalous LMCA from the right coronary sinus. Obstruction can be due to acute angle of take-off or slit-like orifice (1); compression of the intramural segment (2); Lateral luminal compression of the portion between the pulmonary artery and the aorta (3). LMCA: left main coronary artery, RCA: right coronary artery, PA: pulmonary artery, LAD: left anterior descending artery, LCX: left circumflex artery Image created by the authors.

**Figure 11:**
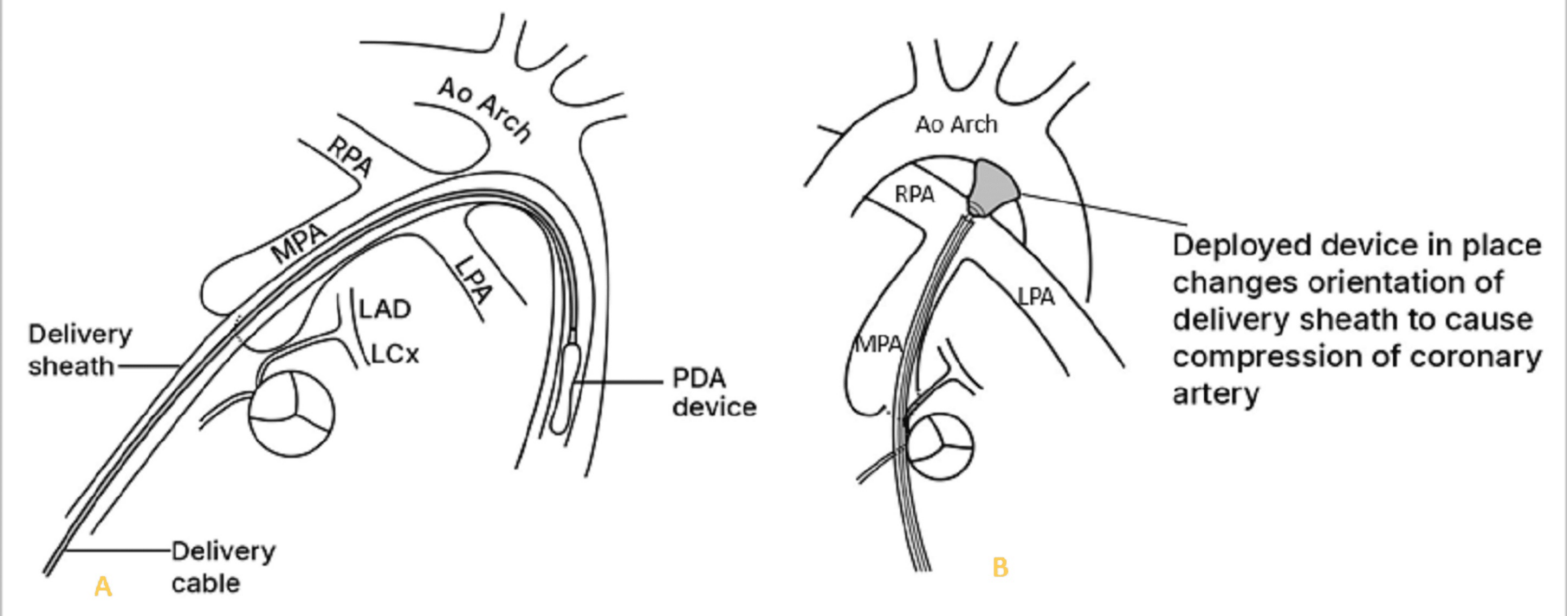
Illustration showing the PDA device and its delivery system. A: Device in the descending aorta, not yet deployed. B: deployed device changed the orientation of the delivery system causing compression of the intramural portion of anomalous left main coronary artery. PDA: patent ductus arteriosus, MPA: main pulmonary artery, Ao: aorta, RPA: right pulmonary artery; LPA: left pulmonary artery, LAD: left anterior descending artery, LCX: left circumflex artery Image created by the authors.

In cases of AOCA from the contralateral aortic sinus (Figure 10), acute angle of take-off, slit-like orifice and compression of the intramural segment by the aortic commissure are all thought to narrow the coronary orifice [4,9]. Lateral luminal compression of the intramural portion between the pulmonary artery and the aorta causes myocardial ischemia, ventricular arrhythmia and sudden cardiac death [3,5,8,9]. This can occur during or just after vigorous physical exertion or transcatheter manipulation in the right ventricular outflow tract [8,9]. In the case under discussion the patient's sudden decline and cardiac arrest was caused by catheter manipulation in the RVOT. The device and its delivery system most likely caused compression of the intramural portion of anomalous left main coronary artery (see illustration in Figure 11). This compromised myocardial perfusion which led to ischemia, ventricular arrhythmia and eventually cardiac arrest. The child had a dramatic improvement upon retrieval of the sheath into the IVC. 

PDA device closure is the treatment modality of choice among children with PDA; it is mostly a safe procedure [7]. Adverse events related to pediatric cardiac catheterization occur in 4-10% of procedures [9]. The Uganda Heart Institute has been conducting cardiac catheterization procedures for close to 13 years. In our settings, it is a relatively safe procedure with about 90% optimal outcome for PDA device closure, and adverse events are seen in about 3.9% of procedures [13]. Many are minor in nature; however, major events can also occur, and may be unpredictable. The major events may be patient- and/or procedure-related, and potentially life-threatening [9,13]. This may result from changes in hemodynamic stability and cardiac output, vessel or cardiac injury, or device embolization. In some studies, cardiac arrest incidence in children with CHD undergoing pediatric catheterization is about 0.2 per 100 procedures [9].

Definitive surgical options for this rare abnormality include coronary translocation and/or coronary artery bypass grafting [14]. Coronary artery stenting to avoid systolic compression has been attempted [14].

## Conclusions

In this case we present a rare cause of cardiac arrest during a routine PDA device closure in a six-year-old female who was later found to have anomalous LMCA from the right aortic sinus of Valsalva. The cardiac arrest in this case was associated with catheter manipulations in the RVOT causing compression of the anomalous LMCA. This case demonstrates that children with AOCA can be asymptomatic or may have symptoms attributable to another underlying congenital cardiac abnormality. She had symptoms pointing mostly to heart failure due to PDA and no features of myocardial ischemia. Therefore, clinicians should always maintain a high index of suspicion for coronary abnormalities in children who sustain cardiac arrest during routine cardiac catheterization. A comprehensive first echocardiography should include coronary artery assessment for all patients going to the catheterization laboratory. The presence of a major cardiac anomaly, a PDA in this case should not drive the focus away from completing a comprehensive echocardiography study.

## References

[1] Kashyap JR, Kumar S, Reddy S (2021). Prevalence and pattern of congenital coronary artery anomalies in patients undergoing coronary angiography at a tertiary care hospital of northern India. Cureus.

[2] Latif A, Khalil MS, Ullah S, Parvez M, Shah HA (2022). Prevalence and pattern of congenital coronary artery anomalies in patients undergoing coronary angiography at a tertiary care hospital. Pak J Med Health Sci.

[3] Roberts WC, Shirani J (1992). The four subtypes of anomalous origin of the left main coronary artery from the right aortic sinus (or from the right coronary artery). Am J Cardiol.

[4] Lee BY (2009). Anomalous right coronary artery from the left coronary sinus with an interarterial course: is it really dangerous?. Korean Circ J.

[5] Molossi S, Martínez-Bravo LE, Mery CM (2019). Anomalous aortic origin of a coronary artery. Methodist Debakey Cardiovasc J.

[6] Molossi S, Agrawal H (2017). Clinical evaluation of anomalous aortic origin of a coronary artery (AAOCA). Congenit Heart Dis.

[7] Lam JY, Lopushinsky SR, Ma IW, Dicke F, Brindle ME (2015). Treatment options for pediatric patent ductus arteriosus: systematic review and meta-analysis. Chest.

[8] Dotan M, Roguin A, Sinyor D (2013). Increased incidence of coronary artery origin anomalies associated with isolated patent ductus arteriosus. Pediatr Cardiol.

[9] Sawasdiwipachai P, Phothong B (2018). Cardiac arrest in pediatric patients with congenital heart diseases undergoing cardiac catheterization: a retrospective study. J Med Assoc Thai.

[10] AlQubbany A, Alqurashi Y, Zagzoog A, Almehmadi F, Al-Husayni F, Ahmad A, Albugami S (2023). Anomalous coronary arteries: a cause for malignant arrhythmias. Cureus.

[11] Imran A, Carter H, Mensah J, Ahmed M, Solano J (2025). Sudden collapse in a child revealing a malignant coronary anomaly: a case of congenital anomaly of left coronary artery origin. Cureus.

[12] Cantinotti M, Giordano R, Assanta N (2021). Echocardiographic screening of anomalous origin of coronary arteries in athletes with a focus on high take-off. Healthcare (Basel).

[13] Mbabazi N, Aliku T, Namuyonga J (2024). Congenital heart disease cardiac catheterization at Uganda Heart Institute, a 12-year retrospective study of immediate outcomes. BMC Cardiovasc Disord.

[14] Hariharan R, Kacere RD, Angelini P (2002). Can stent-angioplasty be a valid alternative to surgery when revascularization is indicated for anomalous origination of a coronary artery from the opposite sinus?. Tex Heart Inst J.

